# Genotoxicity of Marijuana in Mono-Users

**DOI:** 10.3389/fpsyt.2021.753562

**Published:** 2021-12-06

**Authors:** Eunice Fabian-Morales, Carmen Fernández-Cáceres, Adriana Gudiño, Marco A. Andonegui Elguera, Karla Torres-Arciga, Marco Armando Escobar Arrazola, Laura Tolentino García, Yair E. Alfaro Mora, Diego A. Oliva-Rico, Rodrigo E. Cáceres Gutiérrez, Julieta Domínguez Ortíz, Clementina Castro Hernández, Luis A. Herrera Montalvo, David Bruno Díaz-Negrete, Nancy Reynoso-Noverón

**Affiliations:** ^1^Unidad de Aplicaciones Avanzadas en Microscopía (ADMiRA), Instituto Nacional de Cancerología (INCan), Red de Apoyo a la Investigación (RAI), Universidad Nacional Autónoma de México (UNAM), Ciudad de México, México; ^2^Unidad de Investigación Biomédica en Cáncer, Instituto Nacional de Cancerología (INCan)-Instituto de Investigaciones Biomédicas, Universidad Nacional Autónoma de México (UNAM), Ciudad de México, México; ^3^Centro de Integración Juvenil (CIJ), Ciudad de México, México; ^4^Dirección General, Instituto Nacional de Medicina Genómica (INMEGEN), Ciudad de México, México; ^5^Dirección de Investigación, Instituto Nacional de Cancerología, Ciudad de México, México

**Keywords:** marijuana, cannabis, drug consumption, tobacco smokers, DNA damage, γH2AX, micronucleus

## Abstract

Marijuana (*Cannabis* sp.) is among the most recurred controlled substances in the world, and there is a growing tendency to legalize its possession and use; however, the genotoxic effects of marijuana remain under debate. A clear definition of marijuana's genotoxic effects remains obscure by the simultaneous consumption of tobacco and other recreational substances. In order to assess the genotoxic effects of marijuana and to prevent the bias caused by the use of substances other than cannabis, we recruited marijuana users that were sub-divided into three categories: (1) users of marijuana-only (M), (2) users of marijuana and tobacco (M+T), and (3) users of marijuana plus other recreative substances or illicit drugs (M+O), all the groups were compared against a non-user control group. We quantified DNA damage by detection of γH2AX levels and quantification of micronuclei (MN), one of the best-established methods for measuring chromosomal DNA damage. We found increased levels of γH2AX in peripheral blood lymphocytes from the M and M+T groups, and increased levels of MNs in cultures from M+T group. Our results suggest a DNA damage increment for M and M+T groups but the extent of chromosomal damage (revealed here by the presence of MNs and NBuds) might be related to the compounds found in tobacco. We also observed an elevated nuclear division index in all marijuana users in comparison to the control group suggesting a cytostatic dysregulation caused by cannabis use. Our study is the first in Mexico to assess the genotoxicity of marijuana in mono-users and in combination with other illicit drugs.

## Introduction

Marijuana is the most used illicit drug worldwide, and millions of people are exposed to it annually. Different studies have suggested that a direct relationship between marijuana consumption and increased risk to certain types of cancer exists, such as neonatal tumors of the soft tissues or acute myeloid leukemias in the progeny (1–3). Among young individuals, marijuana use has been linked to head and neck cancer; as well as to testicular and lung cancers (4–8). Nonetheless, neither the whole-plant cannabis extract nor the Δ^9^-Tetrahydrocannabinol (THC), the main psychoactive compound in marijuana, have been confirmed as mutagenic or carcinogenic (9–12).

Marijuana smoke, however, consists to a certain extent, of compounds commonly found in cigarette smoke, and that have been identified as carcinogens by the International Agency for Research on Cancer (IARC) (13). Previous studies have shown that marijuana smoke can alter DNA content and induce aneuploidies in human lung explants (14). In addition, exposure to marijuana smoke condensates has been described to generate DNA damage, DNA adducts, reactive oxygen species (ROS) and cellular stress, all of this suggesting once again that marijuana is genotoxic and mutagenic (15–19).

Population studies reported increased levels of DNA damage and chromosome breakages in marijuana users compared to non-user controls (20, 21). Despite these findings, the limited number of epidemiological studies performed so far has yielded contradictory results; further, tobacco consumption is frequently considered a confusing factor; therefore, a definitive link between marijuana consumption and cancer development has not been established (8, 22–25). More studies of individuals consuming *marijuana-only* are necessary to assume reliable conclusions on the association between marijuana and cancer.

In Mexico the prevalence of marijuana consumption in 2011 was of 1.2% for the population aged 12–65 years (26). Since legalization of marijuana consumption for recreational purposes is a possibility, not only in Mexico but also in other countries, the proportion of users can potentially increase as well as the consequences of its consumption, including chronic diseases, such as cancer. Therefore, evaluating the genotoxicity of marijuana and biomonitoring its effects on exposed populations is fundamental for improving its regulation and increasing any required protection from its effects.

Knowing the genotoxic potential of a substance is important to predict its detrimental effects at the cellular and organismal levels. Some of the most commonly applied methods for detection of DNA damage and genotoxic potential of a substance are the presence of γH2AX and the cytokinesis-block micronucleus cytome (CBMNcyt) assay, respectively. Phosphorylation of the Ser-139 residue of the histone variant H2AX, forming γH2AX, is an early cellular response to the induction of DNA double-strand breaks (DSB). Detection of this phosphorylation event has emerged as a highly specific and sensitive molecular marker for monitoring DNA damage initiation and resolution (27). On the other hand, the micronucleus (MN) frequency in peripheral blood lymphocytes (PBL) is one of the best-established biomarkers for studying DNA and chromosomal damage, occurring *in vivo* in humans. Consequently, CBMNcyt assay has been extensively used to identify compounds that significantly impact genomic stability (28). Moreover, MN in PBL has been associated prospectively with an increased risk of cancer (29, 30).

Since the genotoxic effects of marijuana consumption are not completely understood, and this type of information is critical for public policy design, the present study addresses the genotoxic effects of marijuana in Mexican consumers. We analyzed the levels of genomic instability and the presence of DNA damage using the CBMNcyt assay and γH2AX levels in marijuana users. Importantly, we made a clear distinction between marijuana mono-users (M), users of marijuana in combination with tobacco (M+T) and users of other illicit drugs (M+O). This is the first study in which the amount of DNA damage and genomic instability are assessed in PBL of marijuana mono-users in Mexico. Also, evaluation of consumers of other drugs allowed us to obtain a vast panorama on the potential genotoxic damage produced by the consumption of illegal substances.

## Materials and Methods

### Study Population and Definition of Inclusion Criteria

Our study population comprised 201 peripheral blood donors (49 females and 152 males; average age, 22.84 ± 8.59 years and age range 13–68 years). Subjects were distributed in four groups as follows: Group M: users of marijuana-only, also known as mono-users (*n* = *51*); Group M+T: users of marijuana and tobacco (*n* = *52*); Group M+O: users of marijuana and other illicit substances (*n* = *46*), and Group Ctrl: control population composed by non-drug users healthy volunteers (*n* = *52*). Illegal drug consumers were recruited from 12 Juvenile Integration Centers (CIJ) from Mexico City and its Metropolitan area. They were regular patients in their first month of treatment and were selected by convenience sampling. Some of the control subjects were college students, and others were staff members, all of them residents of Mexico City and the Metropolitan area. The eligibility criteria for this study are described in Supplementary Figure 1. Written informed consent was obtained from the participants prior to inclusion in this study. This study was approved by the Ethics Committee of the CIJ and the Instituto Nacional de Cancerología (INCan), México. The study was conducted in accordance with the Declaration of Helsinki and local laws.

### Data Collection

A cross-sectional survey was conducted among the study population, and the collected information is summarized in Table 1. Unhealthy alcohol use was determined according to the AUDIT test developed by the World Health Organization (WHO). Marijuana users were asked about age of initiation of marijuana consumption, time as consumer, the frequency and modes of marijuana use, amount (grams) of marijuana consumed per week and the causes of consumption.

**Table 1:** Demographics of the participants in this study.

	**Group 1,** **Control group**	**Group 2,** **Marijuana monousers (M)**	**Group 3,** **Marijuana + Tobacco (M+T)**	**Group 4,** **Marijuana + other substances (M+O)**	***P***
*n*	52	52	51	46	
Age, P50 (P25–P75)	24 (21–27)	17 (16–21)	20 (17–24)	18 (16–27)	<0.001
Gender		*n* (%)			
Male, *n* (%)	23 (44.23%)	45 (86.54)	43 (84.31)	41 (89.13)	<0.001
Female, *n* (%)	29 (55.77)	7 (13.46)	8 (15.69)	5 (10.87)	
Educational level		*n* (%)			
None	–	–	–	1 (2.17)	<0.001
Elementary school	–	2 (3.85)	1 (1.96)	2 (4.35)	
Middle school	2 (3.85)	18 (34.61)	11 (21.57)	10 (21.74)	
High school	5 (9.61)	25 (48.07)	29 (56.86)	28 (60.87)	
Associate degree	–	–	1 (1.96)	1 (2.17)	
Bachellor degree	36 (69.23)	7 (13.46)	9 (17.65)	4 (8.69)	
Graduate School	9 (17.30)	–	–	–	
BMI		*n* (%)			
Normal	32 (61.54)	38 (73.07)	33 (64.70)	27 (58.69)	0.372
Underweight	1 (1.92)	3 (5.76)	5 (9.80)	6 (13.04)	
Overweight	8 (15.38)	8 (15.38)	11 (21.56)	9 (19.56)	
Obesity	4 (7.69)	2 (3.84)	1 (1.96)	4 (8.69)	
No information	7 (13.46)	1 (1.92)	1 (1.96)	–	
AUDIT score		*n* (%)			
No alcohol consumers	6 (11.54)	18 (34.61)	15 (29.41)	16 (34.78)	<0.001
Low	42 (80.77)	25 (48.07)	25 (49.01)	23 (50)	
Moderate	2 (3.85)	3 (5.77)	1 (1.96)	2 (4.34)	
High	1 (1.92)	0 (0)	–	1 (2.17)	
No answer	1 (1.92)	6 (11.54)	10 (19.6)	4 (8.7)	
Substance use					
Number of illicit drugs used in lifetime (including marijuana), P_50_ (P_25_-P_75_)	0	1 (1–3)	2 (1–3)	3 (2–5)	<0.001
Age at first illicit drug use (Mean ± SD)	NA	14.55 ± 2.25	15.37 ± 2.36	14.28 ± 3.03	0.092
Time recognized as marijuana consumer (months), P50 (P25–P75)	NA	36 (24–60)	48 (24–96)	48 (24–114)	0.053
Frequency of marijuana use, in the last 12 months		*n* (%)			
Daily	NA	30 (57.69)	34 (66.66)	33 (71.73)	0.575
More than once per week	NA	15 (28.84)	13 (25.49)	10 (21.73)	
At least once per week	NA	7 (13.46)	4 (7.84)	3 (6.5)	
Weekly marijuana consumption quantity					
Grams consumed, P50 (P25–P75)	NA	10 (5–18)	7.5 (3–16.25)	10 (3–28)	0.551
Modes of Marijuana use		*n* (%)			
Smoked	NA	34 (65.4)	32 (62.7)	33 (71.7)	0.811
Smoked/ingested	NA	8 (15.4)	8 (15.7)	5 (10.9)	
Smoked/inhaled/ingested	NA	9 (17.3)	11 (21.6)	8 (17.4)	
Ingested	NA	1 (1.9)	–	–	
Approximate duration of inhalation when smoking marijuana					
Time (seconds), P50 (P25–P75)	NA	5 (4–10)	5 (3–10)	6 (5–12.5)	0.089
Pattern of drug consumption		*n* (%)			
Experimental	NA	–	1 (1.96)	–	0.135
Social	NA	5 (9.6)	10 (19.6)	12 (26.08)	
Functional	NA	36 (69.2)	31 (60.78)	21 (45.65)	
Dysfunctional	NA	11 (21.2)	8 (15.68)	13 (28.26)	
No information	NA	–	1 (1.96)	–	

*BMI, body mass index; AUDIT, Alcohol Use Disorders Identification Test; P_50_, 50^th^ percentile; P_25_-P_75_, 25th to 75th percentile range; NA, nor applicable*.

### Instruments

#### Marijuana Use

If participants responded “yes” to whether they had used marijuana during the previous 12 months, we asked the age at which they started or first tried marijuana, and for how long they have been consuming marijuana on a regular basis. To estimate the frequency use, we categorized the options as (1) every day, (2) more than once per week, or (3) at least once per week. We also asked about their main route of administration, with response options included: cannabis cigarettes (smoked), water pipes (inhaled), and mixed with food (ingested), the participants could mark more than one option. If the participants inhaled or smoked marijuana, we asked them to estimate the time of inhalation/smoking per joint/bong.

#### Other Illicit Drugs Use

The use of illicit drugs other than marijuana was determined by asking participants whether they had either used of not used during the previous 30 days or/and during the previous 12 months. Participants with a positive answer to this question were classified in the group M+O. The illicit drugs were categorized as below: (1) cocaine; (2) crack; (3) solvents for sniffing; (4) crystal meth; (5) amphetamines, methamphetamines, and amphetaminsulfate (e.g., dextroamphetamine or benzedrine); (6) hallucinogens, magic mushrooms, psilocybin, peyote, and mescaline; (7) benzodiazepines (e.g., diazepam, clonazepam); (8) heroin.

#### Pattern of Marijuana Use

Regarding characterization of pattern for marijuana use, we considered the following categories: (1) *experimental*, marijuana use on 1 or 2 occasions, without recurrences; (2) *social*, consumption begins to be more regular and framed in leisure contexts, with more people; drug use is not an escape or a solution to a conflict; (3) *functional*, the consumer experiments a sensation of excitement and enjoys the experience that marijuana produces; the frequency and quantity increases; the person can develop physical or psychological dependence; (4) *dysfunctional*, the use of marijuana produces deterioration in the social, biological, and psychological fields; the consumer has a large number of inter and intrapersonal problems, criminal behavior, absenteeism from work, and/or abandonment of leisure activities.

### Preparation of Whole-Blood Cultures

Blood samples (8 ml) were collected in heparinized tubes. After obtention, all blood samples were randomly coded, transported to the laboratory at INCan and processed within 8 h following sampling. Approximately 1 ml of each sample was cultured for 72 h at 37°C in 5 ml of RPMI-1640 culture media (Gibco BRL, Life Technologies SrL, Milano, Italy) supplemented with 15% heat-inactivated fetal calf serum (Gibco BRL, Life Technologies SrL, Milano, Italy) and 2% phytohemagglutinin-M (Gibco BRL, Life Technologies SrL, Milano, Italy). Four parallel cultures of each subject were set up to perform karyotype and cytokinesis-block micronucleus cytome assay.

### Karyotype Analysis

Karyotype analysis was performed in all peripheral blood samples included in this study. Harvesting and GTG-banding were performed according to standard procedures, and karyotyping performed by professional cytogeneticists and described according to the International System for Human Cytogenomic Nomenclature 2020 guidelines (31).

### Cytokinesis-Block Micronucleus Cytome (CBMNcyt) Assays in Peripheral Blood

The CBMNcyt assays were conducted as described by Fenech (32). Briefly, cytochalasin-B (Sigma, Milano, Italy) was used at a final concentration of 3 μg/ml and added to cultures to block cytokinesis after 44 h of incubation. Cells were harvested after 72 h of culture, treated with hypotonic solution (0.1 M KCl) for 4 min and fixed in methanol/acetic acid (3:1, v/v). The fixation step was repeated twice, and the fixed cells were spread onto clean glass slides. Then, they were stained with eosin and methylene blue for 5 min in each solution. All slides were coded and read blind. To determine the intra-individual differences, slides of two parallel cultures of each subject were prepared and evaluated. The slides were analyzed with a light microscope with 400× magnification, and CBMNcyt assay parameters such as micronucleus (MN), nuclear buds (NBUDs), and nucleoplasmic bridges (NPBs) were additionally verified under 1000× magnification. A score was obtained for slides from each duplicate culture from two different analyzers using identical microscopes.

We followed the criteria for the selection of bi-nucleate (BN) cells and identification of CBMNcyt assay parameters as previously published by Fenech (32). In order to determine DNA damage, each slide was analyzed for the total number of MN, NBPs and NBUDs, while BN cells with two nuclei surrounded by cytoplasm and a cell membrane obtained from whole-blood cultures were also scored. The number of MN, NPBs and NBUDs was counted in 1,000 BN cells per subject. The frequency of BN cells containing one or more MN was also determined. The number of mono-, bi-, tri-, tetra-, and multi-nucleated cells per 1,000 viable cells was scored to determine cytostatic effects and the rate of mitotic division in the peripheral blood lymphocytes of all individuals. The nuclear division index (NDI) was calculated with the formula: $NDI=\frac{M_{1}+2M_{2}+3M_{3}+4M_{4}}{N}$, where M_1_-M_4_ represent the numbers of cells with 1–4 nuclei and N is the total number of viable cells scored.

### Flow Cytometry Analysis

Following mononuclear cells isolation, cells were fixed with 10% Formaldehyde during 10 min at RT, followed by permeabilization with 0.1% Triton X100 during 30 min at RT, and blocked with 4% BSA. Cells were stained with anti-CD45-Alexa Fluor 700 (Biolegend, cat. 368514) and anti-γH2AX-Alexa Fluor 488 (Biolegend, cat. 613406) during 30 min, washed with PBS 4%-BSA and resuspended in PBS 4%-BSA. Cells treated with etoposide (100 μM) for 1 h were used as a positive control for DNA damage. Samples were acquired in a FACSCanto II flow cytometer. Data were analyzed using the FlowJo software.

### Statistical Analyses

Descriptive statistics were used for comparing the consumption and sociodemographic features among groups. Mean and standard deviation (SD) or Median and interquartile range (IQR) are reported according variable distribution. Qualitative variables were described as frequency and percentages and analyzed using the Chi-square test for k samples.

Differences between groups were calculated using ANOVA for normal distribution and Kruskal Wallis test for variables with non-normal distribution, followed by student's *t*-test for samples with normal distribution and Mann Whitney U test for non-normal distribution. Bivariate and multivariate analyzes were performed to identify the relationship between the sociodemographic and consumption characteristics with the outcome variables. Statistical analyses were performed with STATA v.14. A difference was considered significant if *p* < 0.05.

## Results

Sociodemographic and consumption habits conceivably linked to marijuana use were assessed in this study (Table 1). Analysis of the demographic composition of our cohort showed that the marijuana consumer groups (M, M+T, and M+O) were on average younger than the CTRL group (18.3 vs. 24 yo) and were mostly composed by males (86.58%). The youngest participants were found in group M (mean age: 17 yo, range: 16–21 yo) and the average age for first-time drug use in all groups oscillates between 14 and 15 years old. Individuals in the marijuana consumer groups have on average, a lower educational level compared to CTRL group (Supplementary Figure 2A). About the pattern of marijuana use, the results revealed that the *functional*, interpreted as the sensation of excitement and fun that marijuana produces, was the principal pattern of consumption (Supplementary Figure 2B), which is not surprising given the average young age of the participants.

Analysis of the consumption habits showed that the timespan of marijuana consumption ranges from 24 months (2 years) to 114 months (9.5 years), with no differences among marijuana consumer groups (*p* > 0.05). In the M group, P_50_ is of 36 months, whereas in the M+T group P_50_ is of 48 months for both marijuana and tobacco use, with IQR 21–84 months regarding tobacco consumption. Comparison of the drug consumption frequency, considering only the last year prior to sampling, showed an almost daily use and no significant differences in the frequency of use among marijuana consumer groups (*p* = 0.57).

Estimates of the amount of marijuana consumed per week showed that the M+T group have the lowest average consumption, which however was not significantly different to other marijuana consumer groups (*p* = 0.55). When the modes of marijuana consumption were assessed the most recurrent was smoked, followed by a combination of smoked/inhaled/ingested, and the least popular was the ingested mode (mentioned only by one participant in group M). The duration in reported seconds of inhalation during smoking was 5 s on average without differences among groups (*p* = 0.089). The number of drugs used in a lifetime was found to vary among groups, as expected the M+O group showed the highest number of drugs consumed throughout life, between 2 and 5 different drugs (*p* < 0.001). Finally, low and no alcohol consumption were found in this cohort according to the AUDIT test (even though 21 participants, mainly consumers, did not answer) (Supplementary Figure 2C).

BMI is the easiest parameter to assess for any physiological alteration related to marijuana consumption. Although marijuana use is commonly associated with increased appetite and likelihood of increased BMI, we did not find significant BMI differences among groups (*p* = 0.372) (Supplementary Figure 2D).

### Marijuana and Tobacco Consumers Displayed the Highest Levels of DNA Damage in PBL

**Figure 1:**
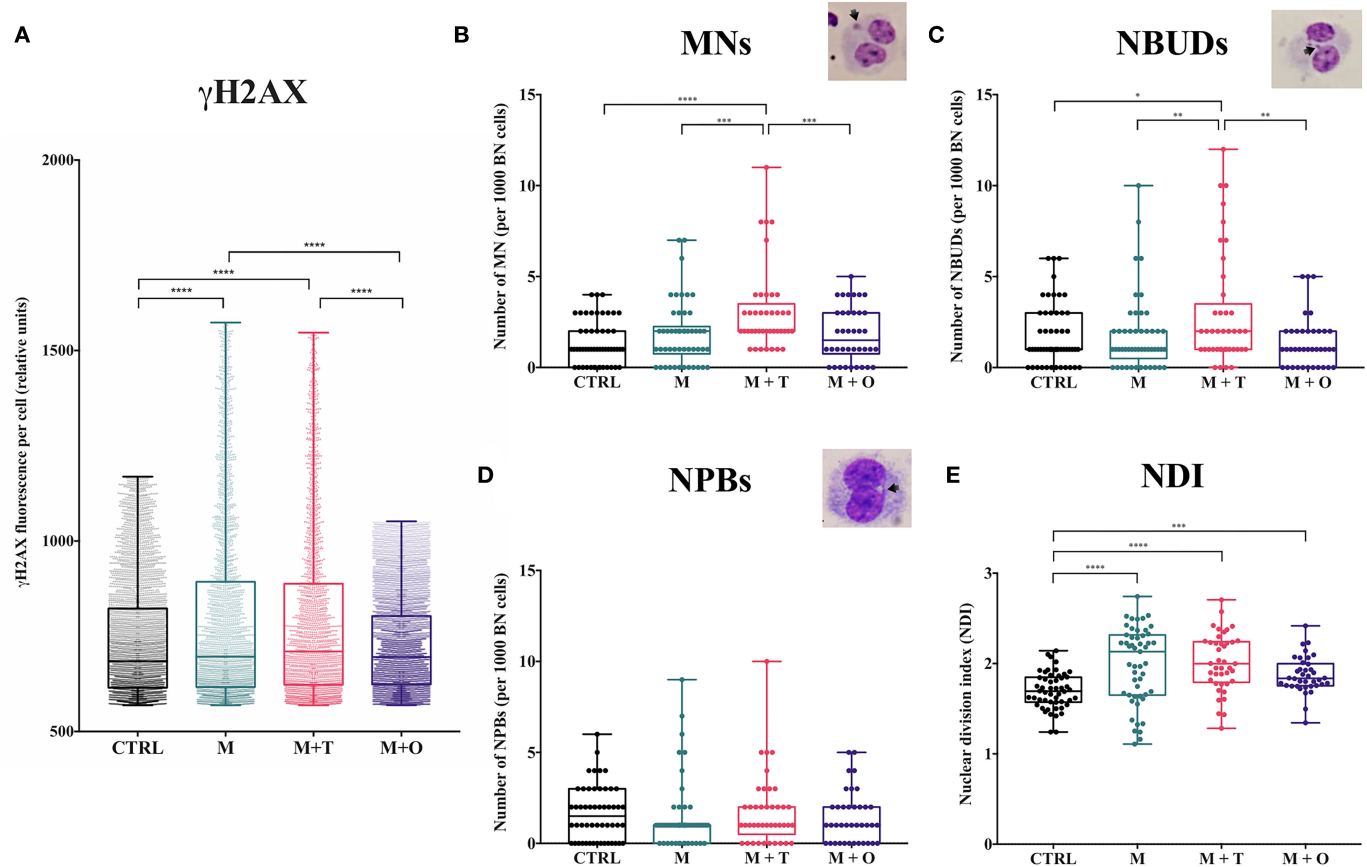
Peripheral blood lymphocytes from marijuana mono-users and in combination with tobacco display the highest levels of genotoxic damage. **(A)** Comparison of the γH2AX fluorescence intensity (FI) *per cell* from freshy isolated lymphocytes. The M and M+T groups show the highest γH2AX FI, without significant differences between them. **(B)** Frequency of micronuclei (MN) in cultured peripheral blood lymphocytes. The highest number of cells with MN was observed in the M+T group in comparison to the other groups. Inset shows a representative binucleated cell with a MN. **(C)** Frequency of nuclear buds (NBUDs) in cultured peripheral blood lymphocytes. The highest frequency of NBUDs was found in the M+T group. Inset shows a representative binucleated cell with an NBUD. **(D)** Frequency of nucleoplasmic bridges (NPBs) in cultured peripheral blood lymphocytes. No differences were observed among groups. Inset shows a representative binucleated cell with an NPB. **(E)** Nuclear division index (NDI) in cultured peripheral blood lymphocytes The NDI is significantly increased in all marijuana users in comparison to the CTRL group. NDI was calculated by quantifying the number of mono, bi, tri, and tetranucleated cells and divided by number of viable cells scored (see Materials and Methods). Error bars indicate mean ± SD; a *p*-value = 0.01–0.05 was considered significant (^*^), a *p* = 0.01 to 0.001 was considered very significant (^**^, ^***^) and a *p* < 0.001 was considered extremely significant (^****^).

Phosphorylation of the H2AX variant (γH2AX) is a highly specific molecular marker of DNA damage (27). γH2AX fluorescence intensity (FI) per cell was assessed in freshly isolated mononuclear cells. As expected, we observed an increase in the levels of γH2AX FI in the M-only and in the M+T groups in comparison to the control group, without significant differences between them (Figure 1A). Unexpectedly, the M+O group has a reduced γH2AX FI with respect to all the other groups.

In order to deepen the genetic toxicology study, we performed the CBMNcyt assay in which T cells were cultured in the presence of Cytochalasin B, and biomarkers as MN, NBUDs and NPBs were scored (Table 2 and Figures 1B–D). The highest number of MN (Figure 1B) and NBUDs (Figure 1C) were found in the M+T group, whereas the number of NPBs did not show significant differences (*p* = 0.138) among groups (Figure 1D).

**Table 2:** DNA damage impact on lymphocytes of marijuana users.

	**Group 1, Control group**	**Group 2, Marijuana monousers (M)**	**Group 3, Marijuana + Tobacco (M+T)**	**Group 4, Marijuana + other substances (M+O)**	***P***
Subjects (*n*)	52	51	51	46	
γH2AX FI per cell in RU (mean)	845.62	1277.56	1282.26	848.91	<0.011^*^
Subjects evaluated for CBMNcyt assay *(n)*	51	46	41	38	
MN (range)/1,000 BN cells	1 (0–2)	2 (1–2.5)	2.5 (2–4)	0.5 (1–3)	<0.001^*^
NBUDs (range)/1,000 BN cells	1 (1–3)	1 (1–2)	2 (1–5)	1 (0–2)	0.014^*^
NPBs (range)/1,000 BN cells	2 (0–3)	1 (0–1)	1 (1–3)	1 (0–2)	0.138
NDI	1.7 (1.2–2.14)	2.01 (1.28–2.22)	1.99 (1.1–2.7)	1.87 (1.3–2.4)	0.033^*^

*FI, Fluorescence intensity; RU, Relative Units; MN, micronucleus; BN cells, bi-nucleated cells; NPBs, nucleoplasmic bridges; NBUDs, nuclear buds; NDI, Nuclear Division Index. The numbers of MN, NPBs and NBUDs were scored on 1,000 BN cells per subject, showing median (Interquartile rank)*.

* *Statistically significant differences by Kruskall Wallis rank sum test*.

Therefore, although both the M-only and the M+T group had increased γH2AX FI, the frequency of the cytogenetic biomarkers were only significantly increased in the M+T group, suggesting increased DNA damage in M and M+T groups but the presence of chromosomal damage could be related only to the compounds found in tobacco.

Assessment of the nuclear division index (NDI) showed that samples from all marijuana consuming groups have a higher NDI in comparison to the cultures from the control group (*p* < 0.0001) (Figure 1E), suggesting that compounds present in marijuana and tobacco might exert an effect on cell proliferation related mechanisms.

Additionally, and given that previous studies have shown that MN frequency tended to be greater in females relative to males (28). The complementary analysis was performed in order to evaluate this possible bias among the population studied, and we did not find statistical differences between the presence of γH2AX neither CBMNcyt assay results by gender within each group (Supplementary Table 1).

### Daily Smoke of Marijuana Increases the Amount of DNA Damage

**Figure 2:**
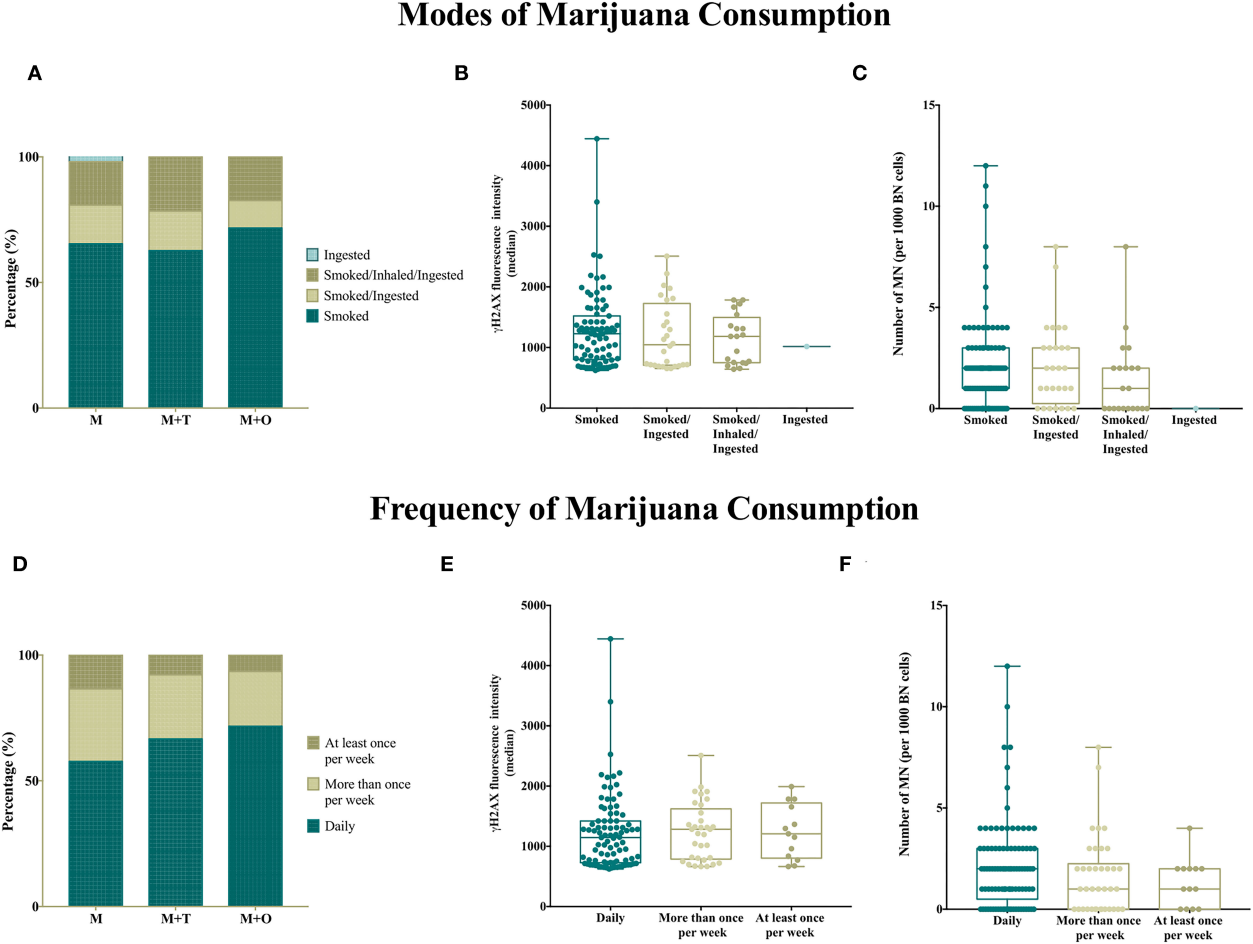
Smoking and daily consumption of marijuana associate with increased genotoxicity. **(A)** Proportion of users reporting different modes of marijuana consumption. “*Smoked*” is the most popular mode of consumption across groups. **(B)** The median fluorescence intensity (FI) of γH2AX is increased in peripheral blood lymphocytes from users that reported “*smoked*” as their mode of marijuana consumption. **(C)** The number of MNs is increased in users who reported “smoked” as the only mode of marijuana use. **(D)** Proportion of users reporting their frequency of marijuana consumption. “*Daily*” consumption is the most common among users. **(E)** The median fluorescence intensity (FI) of γH2AX is increased in peripheral blood lymphocytes from users that reported “*daily*” as their frequency of use. **(F)** The frequency of MN tends to increase when the frequency of marijuana use increases.

We analyzed how the mode of marijuana consumption, as well as the frequency of marijuana consumption, correlate with the levels of γH2AX FI and the frequency of MNs. Importantly, the modes of marijuana consumption followed similar trends in all groups (*p* = 0.811) (Figure 2A), and “*Smoked*” was reported as the most frequently mode of marijuana use. Notably, the levels of γH2AX FI (Figure 2B) and the amount of MNs (Figure 2C) were higher when marijuana was just “*smoked*” in comparison to other modes of consumption.

When the frequency of marijuana consumption was evaluated, no differences among groups were detected (*p* = 0.575), and “*daily*” consumption was the most common answer followed by “*more than once per week*” and “*at least once per week*” (Figure 2D). The highest levels of γH2AX FI (Figure 2E) and a higher frequency of MN (Figure 2F) were observed in the group of people consuming marijuana daily. No direct relationship was found between the amount of MNs or γH2AX FI and the amount of marijuana neither the time as marijuana users. Likewise, neither the additional sociodemographic and consumption characteristics, described in Table 1, were associated significantly with the γH2AX FI nor the CBMNcyt assay results.

### No Chromosomal Aberrations Other Than Rare Constitutional Chromosome Abnormalities Were Observed in Marijuana Users

We discarded chromosome structural or numerical abnormalities in our population of study by performing peripheral blood karyotypes in all the participants (Supplementary Figure 3). Three samples were found to have an altered karyotype, one with a balanced translocation in the M+O group (Supplementary Figure 3A), and two more in the CTRL group, one with a chromosome inversion (Supplementary Figure 3B) and the second one with a chromosome translocation (Supplementary Figure 3C). Constitutional chromosome abnormalities and no association with the consumption of marijuana were determined after analyzing 100 metaphase spreads per donor. Individuals with constitutive chromosome abnormalities received genetic counseling and their samples were excluded from the analysis. Only samples with a normal karyotype were included in the analysis (shown in Table 1 for each group); a normal karyotype is shown in Supplementary Figure 3D. The 46, XY (male) karyotype represented 75.62% of the total population, whereas the 46, XX (female) karyotype represented 24.38%.

## Discussion and Conclusions

The potential genotoxicity of marijuana is still a matter of debate. Previous studies have yielded contradictory results on the effects that marijuana consumption has on human health, therefore, evaluating the effects that marijuana can have on its consuming population is critical. The genotoxicity of marijuana smoke was suggested more than 40 years ago after positive results in a mutagenesis assay (9). However, studies on the effect that cannabinoids have in tumor development and growth, have shown contradictory results, both tumorigenic (33–35) and antitumor (36–40).

Epidemiologic studies have suggested that the continuous use of marijuana is potentially mutagenic (41); nonetheless, the simultaneous consumption of tobacco and/or other illicit drugs, along with marijuana, has hindered definitive conclusions. The present study aimed to evaluate the genotoxic potential of marijuana avoiding the above-mentioned confusing factors. We studied a group of marijuana mono-users (M) that was compared to a group of marijuana and tobacco users (M+T), a group of users that combined marijuana and other illicit drugs, but not tobacco (M+O), and a non-consumer control group. We performed a multidimensional approach that assessed DNA damage, explored modes of marijuana consumption, and analyzed sociodemographic components of marijuana consumption.

The formation of γH2AX is an early cellular response to DSBs induction, and elevated levels of this marker may reflect cancer-associated genomic instability (42). The significant increase of γH2AX FI that we found in PBLs from the M and M+T groups indicates a greater presence of DNA damage in these individuals in comparison to non-consumer controls. Although follow-up studies are required in these populations, continuous induction of DNA damage is a factor that increases the probability of a precancerous lesion (43, 44).

Additionally, and in order to evaluate the presence and the extent of chromosomal damage and cytostasis events in the marijuana users, we performed the CBMNcyt assay. We found a significant increase in the frequency of MN and NBUDs when marijuana is combined with tobacco, but not in the marijuana-only group. These markers are considered as events of genetic damage and may represent a reflection of misrepaired DNA breaks, dysregulation on telomere length as well as malfunctions in the mitotic machinery and DNA amplification (45).

Previous studies tested cannabidiol (CBD), one of the active ingredients of marijuana, found evidence of MNs induction in bone marrow cells of mice at low CBD concentrations (46). In contrast, low doses of THC are usually not associated with genotoxicity (20, 47–49). Interestingly and similar to our results, Souza et al., reported genetic damage in PBLs from marijuana users using a comet assay, and increased MNs in the buccal mucosa of marijuana and tobacco users but not in marijuana mono-users, in comparison to a control group (50). It is important to highlight that the consumption of tobacco *per se* can potentiate the amount of damage, on a dose-dependent fashion (51–53), thus our results suggest that tobacco consumption could potentiate the effects of marijuana.

On the other hand, the unexpected observation that the M+O group, those combining marijuana with other illicit drugs, did not show increased DNA damage could be related to differences in marijuana use not reported in interviews, and also implies that more studies would are needed for defining the genotoxic contribution of every substance consumed in this group.

Interestingly, the mode of consumption and administration of marijuana might be responsible for the discrepancies observed on the reported effects of marijuana (50), and evidence suggest that the protective effects of marijuana are lost when it enters the body through the respiratory tract, similar to tobacco smoke, and then its harmful effects might appear. In fact, marijuana and tobacco smoke show striking similarities in terms of their physical and chemical properties (15, 16, 54), several of them classified as carcinogenic by the IARC (13). Smoking is the preferred mode of marijuana use, and it is estimated that 0.5–1 g of the plant contains 20 mg of THC that is directly consumed as a result of plant combustion (55). Our population of study reported “*Smoke*” as their main mode of consumption. When we compared the γH2AX IF and number of MNs among the different combinations of modes of consumption, we observed more DNA damage when marijuana was smoked. Also, “*daily*” marijuana consumption was associated with increased DNA damage.

Besides the genotoxic potential of marijuana consumption, our study also explores socio-demographic variables, thus giving an extended panorama to our study. Significant associations have been described between the consumers' demographic characteristics and drug dependence. Similar to other studies, our marijuana user population is mainly constituted by male young individuals (56, 57). We found a positive correlation between marijuana consumption and having a low educational level. As previously suggested by other studies, marijuana use during adolescence can have negative long-term effects on school completion (58), however a sampling bias may affect the control group. Contrary to other studies, we did not find a correlation between marijuana consumption and predisposition to alcoholism (59). Marijuana consumption is also commonly associated with increased appetite and body weight gain, however the findings in this area have been inconsistent (60, 61), in our study we did not find such an association, although these observational studies might also be subject of confusing factors (62).

In summary, our results suggest that marijuana use induces DNA DSBs in PBL cells of cannabis users, however the chromosomal damage identified by the frequency of MN and NBUDs is increased only when marijuana is co-used with tobacco. Additionally, all populations of marijuana users showed a significantly increase of the NDI compared to the control group, suggesting that cannabis consumption dysregulates the cell cycle. In this study we recruited mono-users of marijuana, a difficult to accomplish criteria whose lack of fulfillment in previous studies has interfered with a clear definition of marijuana as a DNA damaging agent or a possible carcinogen. This is, to our knowledge, the first study assessing the cytotoxicity of marijuana, alone or in combination with other illicit drugs in the Mexican population. However, its important to mention that more population studies are needed to confirm, extend our findings, and establish a statistically stronger association between marijuana use and cancer.

### Limitations

The present study should be considered in light of its limitations. First, this was a cross-sectional study, which precludes any causal inferences to be drawn from the results. Second, because all data were self-reported, data accuracy cannot be definitively determined. Third, our sample was restricted to patients attending youth integration centers (CIJ), with a small number of patients who use marijuana alone, therefore, our population of marijuana consumers is restricted to young people aged between 16 and 27 years, mostly males. Consequently, caution should be used when extrapolating our findings to women or men of different ages. Further studies will need to examine whether our findings are applicable to female and elder populations.

## Data Availability Statement

The raw data supporting the conclusions of this article will be made available by the authors, without undue reservation.

## Ethics Statement

This study was reviewed and approved by the Research Ethics Committee from the Instituto Nacional de Cancerología (INCan, authorization protocol 017/036/II CEI/1228/17) and Centros de Integración Juvenil (CIJ) from Mexico City. Written informed consent to participate in this study was provided by the participants and in the case of under-aged participants, the consent was authorized by the legal guardian/next of kin.

## Author Contributions

EF-M, CF-C, MA, YA, DO-R, RC, JD, CC, LH, DD-N, and NR-N: conceptualization. EF-M, AG, MA, KT-A, ME, LT, YA, DO-R, RC, JD, and NR-N: methodology. EF-M, AG, KT-A, ME, LT, MA, YA, DO-R, RC, and JD: validation. EF-M, AG, MA, KT-A, ME, LT, YA, DO-R, RC, and CC: project administration. EF-M and NR-N: formal analysis, writing—review and editing, and visualization. CC, LH, DD-N, and NR-N: resources. EF-M, MA, and NR-N: data curation. EF-M and CF-C: writing—original draft. CF-C, LH, DD-N, and NR-N: supervision. NR-N: funding acquisition. All authors contributed to the article and approved the submitted version.

## Funding

This work was supported by Consejo Nacional de Ciencia y Tecnología (CONACYT) through the 2017 funding Fondo Sectorial de Investigación en Salud y Seguridad Social Grant number 290444 PI NR-N.

## Conflict of Interest

The authors declare that the research was conducted in the absence of any commercial or financial relationships that could be construed as a potential conflict of interest.

## Publisher's Note

All claims expressed in this article are solely those of the authors and do not necessarily represent those of their affiliated organizations, or those of the publisher, the editors and the reviewers. Any product that may be evaluated in this article, or claim that may be made by its manufacturer, is not guaranteed or endorsed by the publisher.

## References

[1] Berbel Tornero O Ferrís i Tortajada J Donat Colomer J Ortega García JA Verdeguer Miralles A. Factores de riesgo asociados a los tumores neonatales. Experiencia de una unidad de salud medioambiental pediátrica (PEHSU-Valencia). An Pediatr. (2006) 64:439–48. 10.1157/13087871 16756885

[2] Cancer Incidence and Survival Among Children and Adolescents - Pediatric Monograph - SEER Publications 1975-1995. SEER. Available online at: https://seer.cancer.gov/archive/publications/childhood/index.html (accessed May 3, 2020).

[3] Grufferman S Schwartz AG Ruymann FB Maurer HM. Parents' use of cocaine and marijuana and increased risk of rhabdomyosarcoma in their children. Cancer Causes Control. (1993) 4:217–24. 8318638 10.1007/BF00051316

[4] Gurney J Shaw C Stanley J Signal V Sarfati D. Cannabis exposure and risk of testicular cancer: a systematic review and meta-analysis. BMC Cancer. (2015) 15:897. 10.1186/s12885-015-1905-6 26560314 PMC4642772

[5] Huang Y-HJ Zhang Z-F Tashkin DP Feng B Straif K Hashibe M. An epidemiologic review of marijuana and cancer: an update. Cancer Epidemiol Biomarkers Prev. (2015) 24:15–31. 10.1158/1055-9965.EPI-14-1026 25587109 PMC4302404

[6] Lacson JCA Carroll JD Tuazon E Castelao EJ Bernstein L Cortessis VK. Population-based case-control study of recreational drug use and testis cancer risk confirms an association between marijuana use and nonseminoma risk. Cancer. (2012) 118:5374–83. 10.1002/cncr.27554 22965656 PMC3775603

[7] Daling JR Doody DR Sun X Trabert BL Weiss NS Chen C. Association of marijuana use and the incidence of testicular germ cell tumors. Cancer. (2009) 115:1215–23. 10.1002/cncr.24159 19204904 PMC2759698

[8] Aldington S Harwood M Cox B Weatherall M Beckert L Hansell A. Cannabis use and risk of lung cancer: a case-control study. Eur Respir J. (2008) 31:280–6. 10.1183/09031936.00065707 18238947 PMC2516340

[9] Wehner FC van Rensburg SJ Thiel PG. Mutagenicity of marijuana and Transkei tobacco smoke condensates in the salmonella/microsome assay. Mutat Res Genet Toxicol. (1980) 77:135–42. 10.1016/0165-1218(80)90130-5 6990238

[10] Berryman SH Anderson RA Weis J Bartke A. Evaluation of the co-mutagenicity of ethanol and Δ9-tetrahydrocannabinol with Trenimon. Mutat Res Genet Toxicol. (1992) 278:47–60. 10.1016/0165-1218(92)90285-8 1370119

[11] Van Went GF. Mutagenicity testing of 3 hallucinogens: LSD, psilocybin and delta 9-THC, using the micronucleus test. Experientia. (1978) 34:324–5. 10.1007/BF01923013 631257

[12] Zimmerman S Zimmerman AM. Genetic effects of Marijuana. Int J Addict. (1990) 25:19–33. 10.3109/10826089009067003 2174024

[13] Smith CJ Perfetti TA Garg R Hansch C. IARC carcinogens reported in cigarette mainstream smoke and their calculated log P values. Food Chem Toxicol. (2003) 41:807–17. 10.1016/S0278-6915(03)00021-8 12738186

[14] Leuchtenberger C Leuchtenberger R Ritter U Inui N. Effects of Marijuana and tobacco smoke on DNA and chromosomal complement in human lung explants. Nature. (1973) 242:403–4. 10.1038/242403a0 4701204

[15] Maertens RM White PA Rickert W Levasseur G Douglas GR Bellier PV. The genotoxicity of mainstream and sidestream marijuana and tobacco smoke condensates. Chem Res Toxicol. (2009) 22:1406–14. 10.1021/tx9000286 19947653

[16] Moir D Rickert WS Levasseur G Larose Y Maertens R White P. A comparison of mainstream and sidestream marijuana and tobacco cigarette smoke produced under two machine smoking conditions. Chem Res Toxicol. (2008) 21:494–502. 10.1021/tx700275p 18062674

[17] Sarafian TA Magallanes JA Shau H Tashkin D Roth MD. Oxidative stress produced by marijuana smoke. An adverse effect enhanced by cannabinoids. Am J Respir Cell Mol Biol. (1999) 20:1286–93. 10.1165/ajrcmb.20.6.3424 10340948

[18] Sherman MP Aeberhard EE Wong VZ Simmons MS Roth MD Tashkin DP. Effects of smoking marijuana, tobacco or cocaine alone or in combination on DNA damage in human alveolar macrophages. Life Sci. (1995) 56:2201–7. 10.1016/0024-3205(95)00208-N 7776850

[19] Singh R Sandhu J Kaur B Juren T Steward WP Segerbäck D. Evaluation of the DNA damaging potential of cannabis cigarette smoke by the determination of acetaldehyde derived N2-ethyl-2'-deoxyguanosine adducts. Chem Res Toxicol. (2009) 22:1181–8. 10.1021/tx900106y 19449825

[20] Stenchever MA Kunysz TJ Allen MA. Chromosome breakage in users of marihuana. Am J Obstetr Gynecol. (1974) 118:106–13. 10.1016/S0002-9378(16)33653-5 4808863

[21] Chiesara E Cutrufello R Rizzi R. Chromosome damage in heroin-marijuana and marijuana addicts. Arch Toxicol Suppl. (1983) 6:128–30. 10.1007/978-3-642-69083-9_20 6605134

[22] Hashibe M Morgenstern H Cui Y Tashkin DP Zhang Z-F Cozen W. Marijuana use and the risk of lung and upper aerodigestive tract cancers: results of a population-based case-control study. Cancer Epidemiol Biomarkers Prev. (2006) 15:1829–34. 10.1158/1055-9965.EPI-06-0330 17035389

[23] Sidney S Quesenberry CP Friedman GD Tekawa IS. Marijuana use and cancer incidence (California, United States). Cancer Causes Control. (1997) 8:722–8. 10.1023/A:1018427320658 9328194

[24] Voirin N Berthiller J Benhaïm-Luzon V Boniol M Straif K Ayoub WB. Risk of lung cancer and past use of Cannabis in Tunisia. J Thorac Oncol. (2006) 1:577–9. 10.1097/01243894-200607000-00013 17409920

[25] Sasco AJ Merrill RM Dari I Benhaïm-Luzon V Carriot F. A case-control study of lung cancer in Casablanca, Morocco. Cancer Causes Control. (2002) 13:609–16. 10.1023/A:1019504210176 12296508

[26] Villatoro-Velázquez JA Medina-Mora ME Fleiz-Bautista C Téllez-Rojo MM Mendoza-Alvarado LR Romero-Martínez M. Encuesta Nacional de Adicciones 2011: Reporte de Drogas. Instituto Nacional de Psiquiatría Ramón de la Fuente Muñiz. Instituto Nacional de Salud Pública; Secretaría de Salud (2012). Available online at: http://www.conadic.salud.gob.mx/pdfs/ENA_2011_DROGAS_ILICITAS_.pdf (accessed May 3, 2020).

[27] Mah L-J El-Osta A Karagiannis TC. γH2AX: a sensitive molecular marker of DNA damage and repair. Leukemia. (2010) 24:679–86. 10.1038/leu.2010.6 20130602

[28] Fenech M Bonassi S. The effect of age, gender, diet and lifestyle on DNA damage measured using micronucleus frequency in human peripheral blood lymphocytes. Mutagenesis. (2011) 26:43–9. 10.1093/mutage/geq050 21164181

[29] Bonassi S Znaor A Ceppi M Lando C Chang WP Holland N. An increased micronucleus frequency in peripheral blood lymphocytes predicts the risk of cancer in humans. Carcinogenesis. (2007) 28:625–31. 10.1093/carcin/bgl177 16973674

[30] Pardini B Viberti C Naccarati A Allione A Oderda M Critelli R. Increased micronucleus frequency in peripheral blood lymphocytes predicts the risk of bladder cancer. Br J Cancer. (2017) 116:202–10. 10.1038/bjc.2016.411 27959887 PMC5243995

[31] McGowan-Jordan J Hastings RJ Moore S. ISCN 2020: An International System for Human Cytogenomic Nomenclature. Karger (2020). 10.1159/isbn.978-3-318-06867-2 34407535

[32] Fenech M. Cytokinesis-block micronucleus cytome assay. Nat Protoc. (2007) 2:1084–104. 10.1038/nprot.2007.77 17546000

[33] Zhu LX Sharma S Stolina M Gardner B Roth MD Tashkin DP. Delta-9-tetrahydrocannabinol inhibits antitumor immunity by a CB2 receptor-mediated, cytokine-dependent pathway. J Immunol. (2000) 165:373–80. 10.4049/jimmunol.165.1.373 10861074

[34] Sarafian TA Tashkin DP Roth MD. Marijuana smoke and Delta(9)-tetrahydrocannabinol promote necrotic cell death but inhibit Fas-mediated apoptosis. Toxicol Appl Pharmacol. (2001) 174:264–72. 10.1006/taap.2001.9224 11485387

[35] Russo C Ferk F Mišík M Ropek N Nersesyan A Mejri D. Low doses of widely consumed cannabinoids (cannabidiol and cannabidivarin) cause DNA damage and chromosomal aberrations in human-derived cells. Arch Toxicol. (2019) 93:179–88. 10.1007/s00204-018-2322-9 30341733 PMC6342871

[36] Romano B Borrelli F Pagano E Cascio MG Pertwee RG Izzo AA. Inhibition of colon carcinogenesis by a standardized Cannabis sativa extract with high content of cannabidiol. Phytomedicine. (2014) 21:631–9. 10.1016/j.phymed.2013.11.006 24373545

[37] Aviello G Romano B Borrelli F Capasso R Gallo L Piscitelli F. Chemopreventive effect of the non-psychotropic phytocannabinoid cannabidiol on experimental colon cancer. J Mol Med. (2012) 90:925–34. 10.1007/s00109-011-0856-x 22231745

[38] Massi P Vaccani A Ceruti S Colombo A Abbracchio MP Parolaro D. Antitumor effects of cannabidiol, a nonpsychoactive cannabinoid, on human glioma cell lines. J Pharmacol Exp Ther. (2004) 308:838–45. 10.1124/jpet.103.061002 14617682

[39] McAllister SD Murase R Christian RT Lau D Zielinski AJ Allison J. Pathways mediating the effects of cannabidiol on the reduction of breast cancer cell proliferation, invasion, and metastasis. Breast Cancer Res Treat. (2011) 129:37–47. 10.1007/s10549-010-1177-4 20859676 PMC3410650

[40] Shrivastava A Kuzontkoski PM Groopman JE Prasad A. Cannabidiol induces programmed cell death in breast cancer cells by coordinating the cross-talk between apoptosis and autophagy. Mol Cancer Ther. (2011) 10:1161–72. 10.1158/1535-7163.MCT-10-1100 21566064

[41] Zhang ZF Morgenstern H Spitz MR Tashkin DP Yu GP Marshall JR. Marijuana use and increased risk of squamous cell carcinoma of the head and neck. Cancer Epidemiol Biomarkers Prev. (1999) 8:1071–8. 10613339

[42] Bonner WM Redon CE Dickey JS Nakamura AJ Sedelnikova OA Solier S. GammaH2AX and cancer. Nat Rev Cancer. (2008) 8:957–67. 10.1038/nrc2523 19005492 PMC3094856

[43] Sedelnikova OA Bonner WM. GammaH2AX in cancer cells: a potential biomarker for cancer diagnostics, prediction and recurrence. Cell Cycle. (2006) 5:2909–13. 10.4161/cc.5.24.3569 17172873

[44] Gorgoulis VG Vassiliou L-VF Karakaidos P Zacharatos P Kotsinas A Liloglou T. Activation of the DNA damage checkpoint and genomic instability in human precancerous lesions. Nature. (2005) 434:907–13. 10.1038/nature03485 15829965

[45] Fenech M Kirsch-Volders M Natarajan AT Surralles J Crott JW Parry J. Molecular mechanisms of micronucleus, nucleoplasmic bridge and nuclear bud formation in mammalian and human cells. Mutagenesis. (2011) 26:125–32. 10.1093/mutage/geq052 21164193

[46] Zimmerman AM Raj AY. Influence of cannabinoids on somatic cells *in vivo*. Pharmacology. (1980) 21:277–87. 10.1159/000137442 6252564

[47] Tahir SK Zimmerman AM. Influence of marihuana on cellular structures and biochemical activities. Pharmacol Biochem Behav. (1991) 40:617–23. 10.1016/0091-3057(91)90372-9 1806949

[48] Mon MJ Jansing RL Doggett S Stein JL Stein GS. Influence of Δ9-tetrahydrocannabinol on cell proliferation and macromolecular biosynthesis in human cells. Biochem Pharmacol. (1978) 27:1759–65. 10.1016/0006-2952(78)90553-1 708456

[49] Henrich RT Nogawa T Morishima A. *In vitro* induction of segregational errors of chromosomes by natural cannabinoids in normal human lymphocytes. Environ Mutagenesis. (1980) 2:139–47. 10.1002/em.2860020206 6276167

[50] Souza DVD Claudio SR Da Silva CLF Marangoni KP Peres RC Ribeiro DA. Genomic instability in peripheral blood and buccal mucosal cells of Marijuana smokers: the impact of tobacco smoke. Asian Pac J Cancer Prev. (2020) 21:1235–9. 10.31557/APJCP.2020.21.5.1235 32458627 PMC7541859

[51] Metgud R Neelesh BT. Effect of staining procedures on the results of micronucleus assay in the exfoliated buccal mucosal cells of smokers and nonsmokers: a pilot study. J Cancer Res Ther. (2018) 14:372–6. 10.4103/0973-1482.157351 29516922

[52] Nersesyan A Kundi M Atefie K Schulte-Hermann R Knasmüller S. Effect of staining procedures on the results of micronucleus assays with exfoliated oral mucosa cells. Cancer Epidemiol Biomarkers Prev. (2006) 15:1835–40. 10.1158/1055-9965.EPI-06-0248 17035390

[53] Nersesyan AK Ellahueñe MF. Measuring DNA damage among smokers. Cancer Epidemiol Biomarkers Prev. (2005) 14:1355. 10.1158/1055-9965.EPI-04-0691 15894704

[54] Graves BM Johnson TJ Nishida RT Dias RP Savareear B Harynuk JJ. Comprehensive characterization of mainstream marijuana and tobacco smoke. Sci Rep. (2020) 10:7160. 10.1038/s41598-020-63120-6 32345986 PMC7188852

[55] Bonfá L Vinagre RC de Figueiredo NV. Cannabinoids in chronic pain and palliative care. Revista Brasileira de Anestesiologia. (2008) 58:267–79. 10.1590/s0034-70942008000300010 19378523

[56] Agrawal A Lynskey MT. Does gender contribute to heterogeneity in criteria for cannabis abuse and dependence? Results from the national epidemiological survey on alcohol and related conditions. Drug Alcohol Depend. (2007) 88:300–7. 10.1016/j.drugalcdep.2006.10.003 17084563 PMC1905146

[57] Tu AW Ratner PA Johnson JL. Gender differences in the correlates of adolescents' cannabis use. Subst Use Misuse. (2008) 43:1438–63. 10.1080/10826080802238140 18696378 PMC2562034

[58] Beverly HK Castro Y Opara I. Age of first Marijuana use and its impact on education attainment and employment status. J Drug Issues. (2019) 49:228–37. 10.1177/0022042618823007 31341332 PMC6655417

[59] Tarter RE Vanyukov M Kirisci L Reynolds M Clark DB. Predictors of Marijuana use in adolescents before and after licit drug use: examination of the gateway hypothesis. AJP. (2006) 163:2134–40. 10.1176/ajp.2006.163.12.2134 17151165

[60] Rodondi N Pletcher MJ Liu K Hulley SB Sidney S Coronary Artery Risk Development in Young Adults (CARDIA) Study. Marijuana use, diet, body mass index, and cardiovascular risk factors (from the CARDIA study). Am J Cardiol. (2006) 98:478–84. 10.1016/j.amjcard.2006.03.024 16893701

[61] Matthew Warren BS Kimberly Frost-Pineda MPH Mark Gold. Body mass index and Marijuana use. J Addict Dis. (2005) 24:95–100. 10.1300/J069v24n03_08 16186086

[62] Alayash Z Nolde M Meisinger C Baurecht H Baumeister S-E. Cannabis use and obesity-traits: a Mendelian randomization study. Drug Alcohol Depend. (2021) 226:108863. 10.1016/j.drugalcdep.2021.108863 34304124

