# Supplementary figures and images for: Genotoxicity of Marijuana in Mono-Users
Source: Front Psychiatry. 2021 Dec 6;12:753562. doi: 10.3389/fpsyt.2021.753562 (PMC8685240; doi:10.3389/fpsyt.2021.753562)

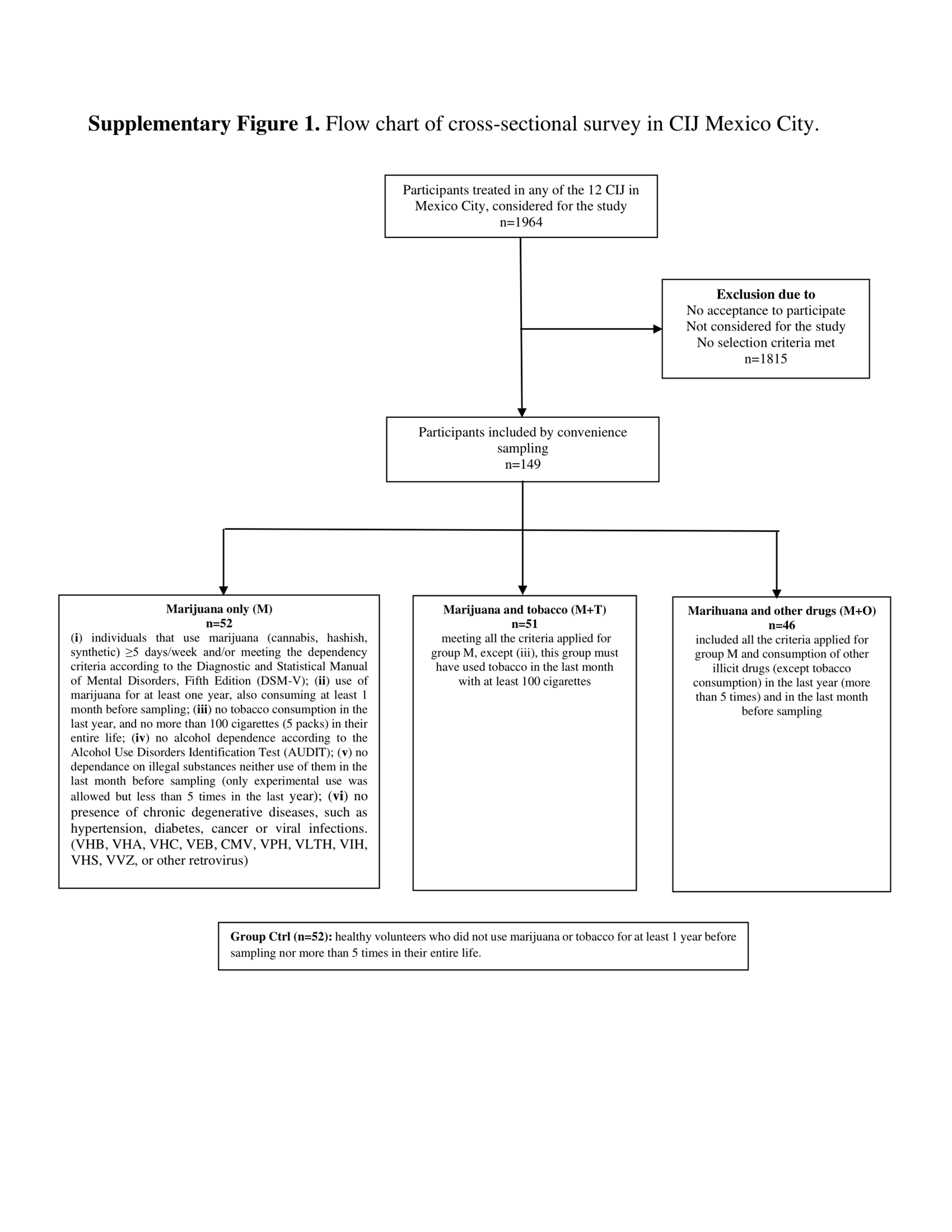

Supplement: Supplementary file 1 [file Image_1.TIFF]

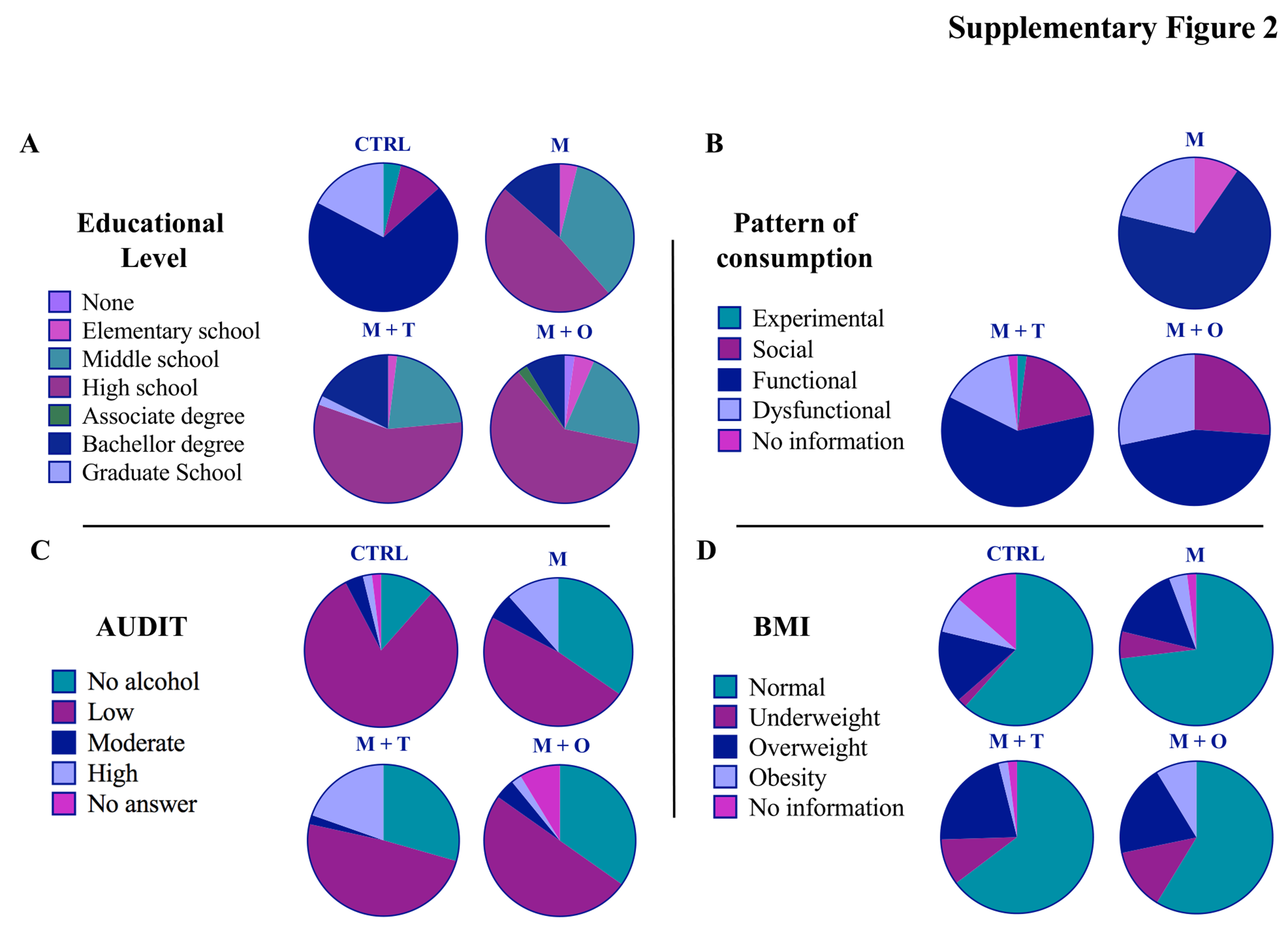

Supplement: Supplementary file 2 [file Image_2.TIFF]

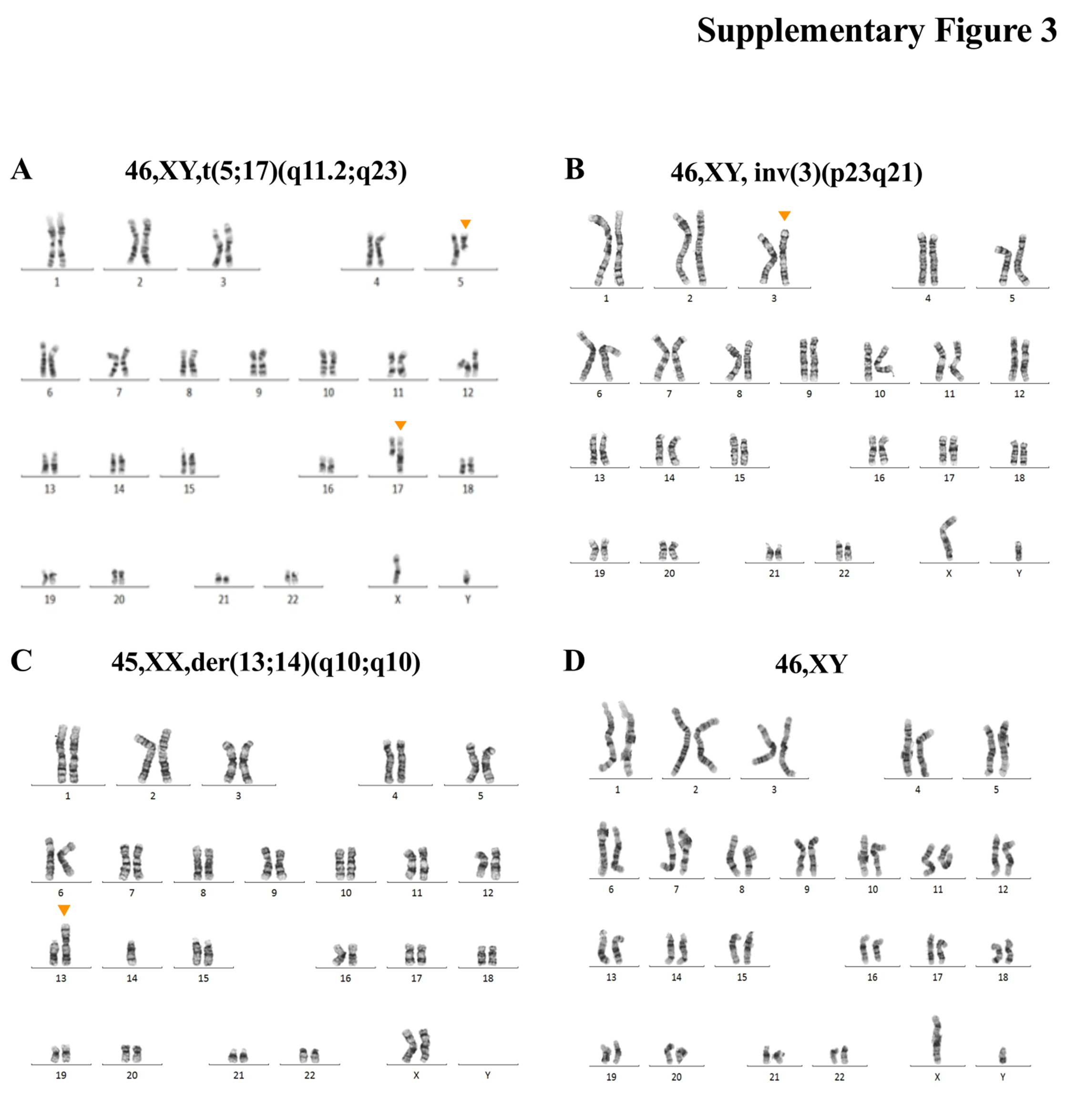

Supplement: Supplementary file 3 [file Image_3.TIFF]
