# Supplementary material for: Genotoxicity of Marijuana in Mono-Users
Source: Front Psychiatry. 2021 Dec 6;12:753562. doi: 10.3389/fpsyt.2021.753562 (PMC8685240; doi:10.3389/fpsyt.2021.753562)
Supplement: Supplementary file 4 [file Table_1.pdf]

Supplementary Table 1. DNA damage impact on lymphocytes of marijuana users differenced by gender in each group

	**Group 1** Control group		**Group 2** Marijuana monousers (M)		**Group 3** Marijuana + Tobacco (M+T)		**Group 4** Marijuana + other substances (M+O)	
**Gender**	M	F	M	F	M	F	M	F
**γH2AX FI per cell in RU (median)**	822.42	914.15	1289.65	939.78	1282.26	1044.60	836.83	876.06
**MN / 1000 BN cells**	1(0-2)	1(1-3)	2(1-3)	0(0-2)	2(2-3)	3.5(2-4)	1(0-3)	3(2-3)
**NBUDs/ 1000 BN cells**	1 (1-2)	1.5(0.5-4)	2(1-2)	1(0-3)	2 (1-6)	1.5(.5-2)	0(1-2)	0(0-2)
**NPBs / 1000 BN cells**	2(0-3)	1.5(0-2.5)	1(0-1)	1(0-3)	1.5(1-3)	1(1-2.5)	1(0-2)	1(1-1)
**NDI**	1.6 (1.5-1.7)	1.6 (1.5-1.8)	2.1 (1.6-2.3)	1.8 (1.6-2.2)	1.9 (1.7-2.2)	2.2 (1.6-2.3)	1.8 (1.7-1.9)	1.9 (1.7-1.9)

FI, Fluorescence intensity; RU, Relative Units; MN, micronucleus; BN cells, bi-nucleated cells; NPBs, nucleoplasmic bridges; NBUDs, nuclear buds; NDI, Nuclear Division Index. The numbers of MN, NPBs and NBUDs were scored on 1000 BN cells per subject, showing median (Interquartile rank). *Statistically significant differences ($p \leq 0.05$) by gender (male/female) in each group by the Mann-Whitney *U* test were analyzed. No statistically significant differences were found.
